# 

**Published:** 1893-02

**Authors:** 


### LITERARY.

ÆDŒOLOGY.—A TREATISE ON GENERATIVE LIFE. St. Clair Publishing Co., New York.

The above is the title of a 4to of 260 pp, on the "Hygiene and Physiology of Generative Life." By Sidney Barrington Elliot, M. D., of Louisville, Ky. The dedication is in these words. "To all who wish to bring into the world healthy children, physically and mentally, and live pure, natural lives, this book is dedicated."

The work is in three parts, and abounds with citations illustrative of the points maintained in the text. As an ethical and hygienic guide from A to Z, it is sure to take rank as a valuable addition to this class of literature.

SECOND AND THIRD ANNUAL REPORTS OF THE STATE BOARD OF HEALTH OF FLORIDA.

We are indebted to Joseph Y. Porter, M.D., State Health Officer, for the above documents, giving a tabular statement by months of the number and causes of death in that far off land of flowers, wherein to our surprise, consumption holds the lead, followed closely in the race by typhoid fever.

TWENTY-SECOND ANNUAL REPORT OF THE MIDDLETOWN STATE HOMEOPATHIC HOSPITAL, Middletown, N. Y.

We are indebted to Dr. Selden H. Talcott, Medical Superintendent, for the above report of this admirable institution, admirably sustained in all its departments.

---

THE ANNUAL REPORT OF THE POST-MASTER GENERAL for the fiscal year ending with June, 1892, is a State document of unusual interest. We learn from it it that the Money Order business transacted through the various branch offices has grown to be an institution of vast magnitude. There were issued during the fiscal year 12,000,000 domestic money orders, aggregating over $120,000,000 in amount, and an aggregate in value of more than $15,000,000 of foreign money orders.

---

We have received from the publishers the following contributions in music:

COLUMBIAN EXPOSITION MARCH.—An original composition by Wm. Rohlfling & Sons, Milwaukee, Wis., which our musical critic says is not without merit. He commends it for its simple harmony and omission of that species of musical gymnastics which tears a passion into a jumble of contending demi-semiquavers, and ends in a kind of discordant row.

TRIFETS' MONTHLY GALAXY OF MUSIC FOR JANUARY, 1893.—Trifets' is a publishing house located at 408 Washington street, Boston, whose aim is to furnish music for the million, at prices which the million can afford to pay. Send 25 cents and get $3.00 worth of instrumental or vocal music, or both.

